# Intracellular Molecular Differences in Aldosterone- Compared to Cortisol-Secreting Adrenal Cortical Adenomas

**DOI:** 10.3389/fendo.2016.00075

**Published:** 2016-06-27

**Authors:** Eric Seidel, Ute I. Scholl

**Affiliations:** ^1^Department of Nephrology, University Hospital Düsseldorf, Heinrich Heine University, Düsseldorf, Germany

**Keywords:** *KCNJ5*, *CACNA1D*, *ATP1A1*, *ATP2B3*, *CTNNB1*

## Abstract

The adrenal cortex is a major site of steroid hormone production. Two hormones are of particular importance: aldosterone, which is produced in the zona glomerulosa in response to volume depletion and hyperkalemia, and cortisol, which is produced in the zona fasciculata in response to stress. In both cases, acute stimulation leads to increased hormone production, and chronic stimulation causes hyperplasia of the respective zone. Aldosterone- and cortisol-producing adenomas (APAs and CPAs) are benign tumors of the adrenal cortex that cause excess hormone production, leading to primary aldosteronism and Cushing’s syndrome, respectively. About 40% of the APAs carry somatic heterozygous gain-of-function mutations in the K^+^ channel *KCNJ5*. These mutations lead to sodium permeability, depolarization, activation of voltage-gated Ca^2+^ channels, and Ca^2+^ influx. Mutations in the Na^+^/K^+^-ATPase subunit *ATP1A1* and the plasma membrane Ca^2+^-ATPase *ATP2B3* similarly cause Na^+^ or H^+^ permeability and depolarization, whereas mutations in the Ca^2+^ channel *CACNA1D* directly lead to increased calcium influx. One in three CPAs carries a recurrent gain-of-function mutation (L206R) in the *PRKACA* gene, encoding the catalytic subunit of PKA. This mutation causes constitutive PKA activity by abolishing the binding of the inhibitory regulatory subunit to the catalytic subunit. These mutations activate pathways that are relatively specific to the respective cell type (glomerulosa versus fasciculata), and there is little overlap in mutation spectrum between APAs and CPAs, but co-secretion of both hormones can occur. Mutations in *CTNNB1* (beta-catenin) and *GNAS* (Gsα) are exceptions, as they can cause both APAs and CPAs through pathways that are incompletely understood.

## Introduction

**Figure 1:**
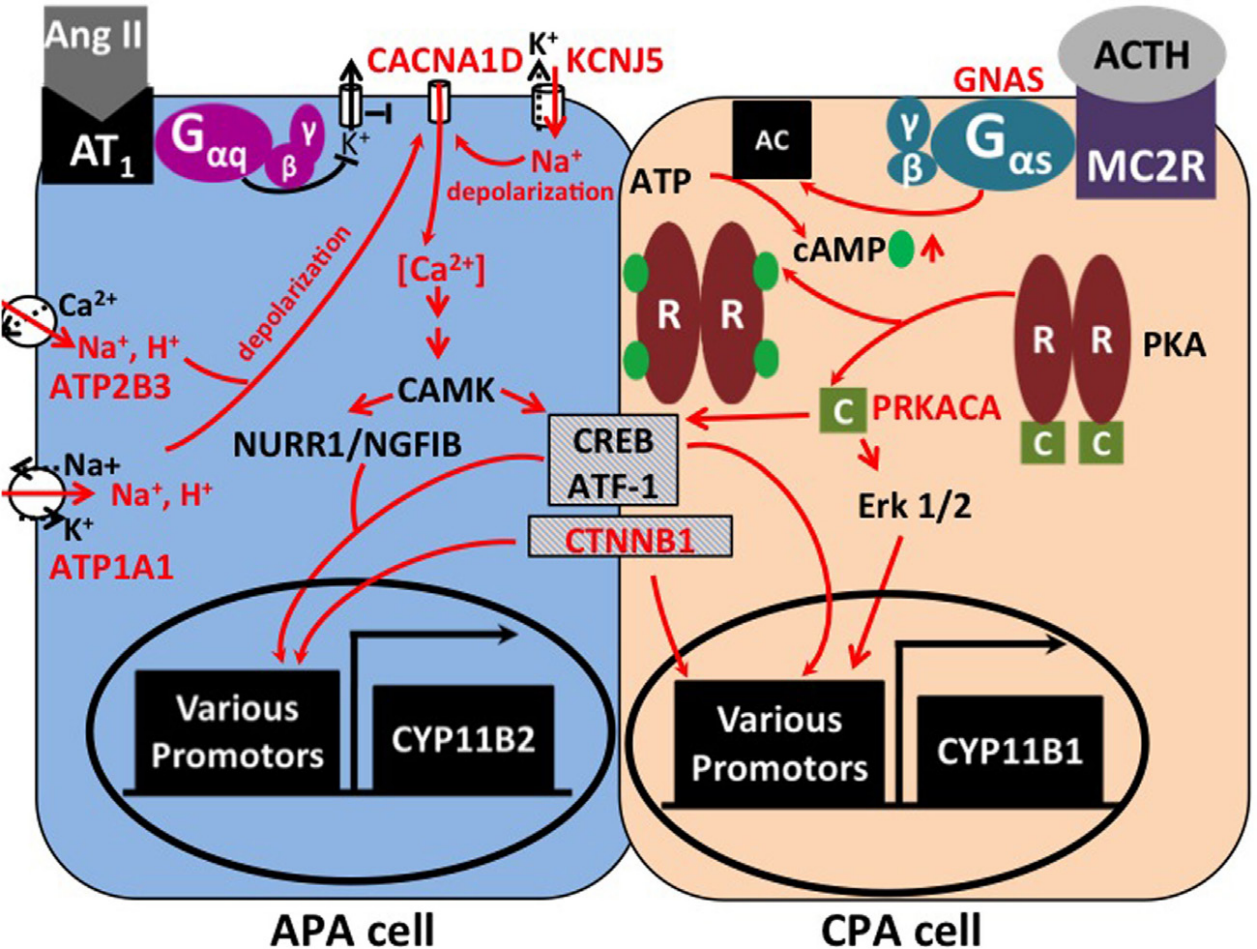
**Signaling pathways affected by mutations in APAs and CPAs**. In zona glomerulosa, binding of angiotensin II (AngII) to its receptor inhibits potassium channels *via* G protein signaling. This leads to depolarization and opening of voltage-gated calcium channels. Increased intracellular calcium results in the activation of Ca^2+^/calmodulin-dependent protein kinase (CAMK) and the activation of transcription factors, such as NURR1/NGFIB, CREB, and ATF-1. As a consequence, genes involved in proliferation and aldosterone production (e.g., aldosterone synthase, *CYP11B2*) are activated (5). Mutations in *KCNJ5, ATP1A1*, and *ATP2B3* lead to abnormal permeability for sodium or protons, which causes cellular depolarization and activation of the same pathways. Similarly, mutations in the calcium channel gene *CACNA1D* lead to increased calcium influx. In the zona fasciculata, binding of corticotropin (ACTH) to the melanocortin receptor (MC2R) causes activation of adenylate cyclase (AC) by the G_αs_ subunit (encoded by *GNAS*). Binding of cAMP to the regulatory subunit (“R”) of protein kinase A (PKA) leads to release of the catalytic subunit (“C,” encoded by *PRKACA*) from the complex. Transcription factors CREB, ATF-1, and Erk 1/2 cause increased expression of genes involved in proliferation and cortisol production, such as 11β-hydroxylase (*CYP11B1*). Hypercortisolism can occur due to activating mutations in *GNAS* and *PRKACA*. Activating mutations in β-catenin (*CTNNB1*) are found in both APAs and CPAs; the underlying mechanisms are incompletely understood.

Adrenal masses are common tumors in humans. Adrenal incidentalomas may be found in more than 4% of computed tomography series (1), and about 7% are malignant (2). Among hormone-producing lesions, besides pheochromocytomas, cortisol-producing and aldosterone-producing adenomas (CPAs and APAs) of the adrenal cortex are frequently diagnosed (2). Aldosterone and cortisol are physiologically synthesized in the two outer layers of the adrenal cortex (zonae glomerulosa and fasciculata, respectively) from their common precursor cholesterol. The two main stimuli of aldosterone production are angiotensin II (ATII) and hyperkalemia. ATII levels rise in states of volume depletion, *via* activation of the renin–angiotensin system. Binding of ATII to the AT1 receptor, a G protein-coupled receptor in the glomerulosa membrane, leads to the inhibition of potassium channels, depolarization and activation of voltage-gated calcium channels, and the release of calcium from intracellular stores (Figure 1). Other factors that physiologically regulate aldosterone release in concert with ATII and K^+^ are corticotropin (ACTH, stimulatory) and atrial natriuretic peptide (ANP, inhibitory) (3). Binding of aldosterone to the mineralocorticoid receptor leads to the increased activity of downstream effectors, such as the Na^+^/K^+^-ATPase or the epithelial sodium channel (ENaC) (4). The increased activity of these pumps and channels in kidney and intestine causes increased sodium and water reabsorption and an increase in systemic blood pressure.

Cortisol is released from the zona fasciculata upon stimulation by pituitary ACTH, in response to stress. ACTH binds to the melanocortin receptor 2, a G protein-coupled receptor, which activates adenylate cyclase (6). As a result, cAMP is produced, which binds to the regulatory subunit of protein kinase A (PKA), causing release of its catalytic subunit. The catalytic subunit then phosphorylates target proteins, such as CREB and ATF, which lead to cortisol production and proliferation (7) (Figure 1).

Cortisol influences a variety of biological processes, including skeletal growth, immune response, glucose and lipid metabolism, cognition, and reproduction (8–10).

Cortisol-producing adenomas and APAs feature the compelling combination of both hormone production and proliferation, suggesting that they carry genetic changes that activate both processes. Such changes have been identified over the past 5 years through exome sequencing. Comparing DNA sequences from tumor specimens and corresponding normal tissue (such as blood or adjacent tissue) can reveal tumor-specific (somatic) mutations, which are candidates for disease causation (11). This review will discuss recent genetic discoveries in APAs and CPAs and the underlying pathways.

## *KCNJ5* Mutations in Primary Aldosteronism

Primary aldosteronism (PA) features autonomous production of aldosterone from the adrenal gland and accounts for about 10% of hypertension in referral centers. The two most common causes are APAs and bilateral adrenal hyperplasia. Other causes, such as unilateral hyperplasia, malignant tumors, or familial hyperaldosteronism, are rare (12–16).

In the first exome sequencing study of APAs, Choi et al. analyzed four tumors and corresponding blood samples (11). This revealed only two to three somatic mutations per tumor. One gene (*KCNJ5*) was mutated in two tumors, with one tumor carrying a heterozygous G151R mutation, and the second carrying a heterozygous L168R mutation. By Sanger sequencing, these two mutations were found in 6 of 18 additional APAs. *KCNJ5* encodes an inward rectifier potassium channel, Kir3.4, or GIRK4. The G151 and L168 residues are located within or close to the selectivity filter of the channel (17), which allows only potassium, but not the smaller sodium ions, to pass through the channel. This suggested an effect of the variants on potassium selectivity. Accordingly, by electrophysiology, mutant channels were found to be permeable to sodium and cause cellular depolarization. These effects were inferred to contribute to aldosterone production and proliferation through the activation of voltage-gated calcium channels and calcium entry (3, 11) (Figure 1). Additional support for the notion that *KCNJ5* mutations are sufficient to cause aldosterone production and proliferation came from the discovery of heterozygous germ line *KCNJ5* mutations in families with early-onset PA and massive bilateral adrenal hyperplasia (11, 18–20). The high frequency of *KCNJ5* mutations in APAs (about 35% in European cohorts, more than 60% in Asian cohorts) has subsequently been confirmed in large cohorts (21–29) (Table 1). A higher prevalence in Asian cohorts may be due to selection bias; individuals with *KCNJ5* mutations tend to have a more florid presentation at least in some cohorts. Interestingly, *KCNJ5* mutations are more prevalent in females than in males, which could account for the higher overall prevalence of APAs in females, a finding that remains unexplained. *In vitro* studies in the aldosterone-producing human adrenocortical cancer cell line HAC15 have demonstrated that gain-of-function mutations in *KCNJ5* lead to increased expression of aldosterone synthase and increased aldosterone production (30–32). Lastly, a recent study confirmed the role of *CYP11B2* transcriptional regulators NURR1 and ATF2 in mutant *KCNJ5*-induced aldosterone production (33) (Figure 1).

**Table 1:** **Mutation frequencies in APAs, A/CPAs, and CPAs**.

Reference	*N*	APA						A/CPA		CPA		
		*CACNA1D*	*KCNJ5*	*ATP2B3*	*ATP1A1*	*GNAS*	*CTNNB1*	*KCNJ5*	*GNAS*	*GNAS*	*PRKACA*	*CTNNB1*
Beuschlein et al. (66)	99	–	–	–	–	–	–	–	–	N/A	22.2	N/A
Goh et al. (69)	55	–	–	–	–	–	–	–	–	5.5	23.6	16.4
Cao et al. (67)	87	–	–	–	–	–	–	–	–	N/A	65.5	N/A
Sato et al. (68)	65	–	–	–	–	–	–	–	–	16.9	52.3	N/A
Di Dalmazi et al. (71)	100	–	–	–	–	–	–	–	–	N/A	22.0	N/A
Thiel et al. (41)	52	–	–	–	–	–	–	–	–	7.7	23.1	25.0
Thiel et al. (41)	4	–	–	–	–	–	–	50.0	NA	–	–	–
Yamada et al. (40)	3	–	–	–	–	–	–	66.7	NA	–	–	–
Nakajima et al. (42)	10	–	–	–	–	–	–	60.0	20.0	–	–	–
Xekouki et al. (84)	53	N/A	30.2	N/A	N/A	N/A	N/A	–	–	–	–	–
Taguchi et al. (28)	23	N/A	65.2	N/A	N/A	N/A	N/A	–	–	–	–	–
Kitamoto et al. (85)	108	1.9	69.4	2.8		N/A	N/A	–	–	–	–	–
Boulkroun et al. (86)	380	N/A	33.9	N/A	N/A	N/A	N/A	–	–	–	–	–
Azizan et al. (87)	73	N/A	41.1	N/A	N/A	N/A	N/A	–	–	–	–	–
Cheng et al. (88)	69	N/A	37.7	N/A	N/A	N/A	N/A	–	–	–	–	–
Kuppusamy et al. (89)	195	N/A	24.6	N/A	N/A	N/A	N/A	–	–	–	–	–
Zheng et al. (27)	168	0.6	76.8	0.6	2.4	N/A	N/A	–	–	–	–	–
Scholl et al. (36)	97	10.3	37.1	3.1	8.2	N/A	2.1	–	–	–	–	–
Scholl et al. (45)	64	7.8	32.8	3.1	1.6	N/A	3.1	–	–	–	–	–
Nakajima et al. (42)	33	N/A	72.3	N/A	N/A	6.1	N/A	–	–	–	–	–
Beuschlein et al. (47)	308	N/A	38.3	1.6	5.2	N/A	N/A	–	–	–	–	–
Williams et al. (24)	112	N/A	39.3	0.9	6.3	N/A	N/A	–	–	–	–	–
Akerstrom et al. (22)	348	N/A	45.1	N/A	N/A	N/A	N/A	–	–	–	–	–
Fernandes-Rosa et al. (23)	474	9.3	38.0	1.7	5.3	N/A	N/A	–	–	–	–	–
Akerstrom et al. (83)	198	1.5	46.5	1.5	3.0	N/A	5.1	–	–	–	–	–
Hong et al. (29)	66	0.0	71.2	0.0	0.0	N/A	N/A	–	–	–	–	–
Wu et al. (25)	148	0.0	59.5	0.7	1.4	N/A	N/A	–	–	–	–	–

*N, number of study subjects; N/A, not available*.

## *KCNJ5* Mutations and Glucocorticoids

Interestingly, tumors with *KCNJ5* mutations tend to be larger than other tumors and have fasciculata-like features by histopathology and gene expression analysis, which may have implications for the radiological diagnosis of these tumors (34–36). Another line of evidence pointing to a more fasciculata-like or mixed glomerulosa–fasciculata phenotype of *KCNJ5*-positive APAs is the finding that heterologous expression of a *KCNJ5* variant in HAC15 cells causes not only upregulation of *CYP11B2* expression but also increased expression of *CYP11B1* and synthesis of hybrid steroids 18-hydroxycorticsol and 18-oxocortisol, as well as corticosterone (31, 33). This raises the question whether *KCNJ5*-positive APAs produce clinically relevant amounts of glucocorticoids. Interestingly, hypersecretion of cortisol and aldosterone are not mutually exclusive in adrenal adenomas, and cases of aldosterone and cortisol co-secreting adenomas (A/CPAs) have been reported (37–40). This phenotype may be underdiagnosed due to incomplete screening for subclinical Cushing’s syndrome (CS) in patients with APAs; many of these patients will not receive dexamethasone suppression tests. Yamada et al. reported three female patients with hypertension and hypokalemia who were diagnosed with A/CPAs. Two had *KCNJ5* mutations (G151R and L168R) (40). Thiel et al. reported *KCNJ5* mutations (G151R and L168R) in two of four A/CPAs, and no mutations in *PRKACA, ATP1A1, ATP2B3*, and *CACNA1D* were found (41). Lastly, Nakajima et al. demonstrated *KCNJ5* mutations in 6 of 10 A/CPAs (42). This suggests that *KCNJ5* mutations may cause excess secretion of not only aldosterone but also glucocorticoids, leading to PA with discrete features of CS. Potential explanations include the overlapping role of transcriptional regulators CREB and ATF in the regulation of both aldosterone and cortisol production (Figure 1) as well as a potential role of Ca^2+^ in cAMP formation (43).

In summary, *KCNJ5* mutations have been extensively studied in the context of PA. However, the physiological role of *KCNJ5* in human adrenal glomerulosa remains largely undetermined, and animal studies have been hampered by extremely low or absent expression of *kcnj5* in rodents (44).

## *CACNA1D* Mutations in Primary Aldosteronism

The gene with the second highest somatic mutation burden in APAs is *CACNA1D*, with frequencies of about 8–11% described in the initial exome sequencing studies and similar findings in a large follow-up study (23, 35, 45) (Table 1). Similar to *KCNJ5* mutations, *CACNA1D* mutations are heterozygous. However, mutations are more scattered throughout the protein. *CACNA1D* encodes an L-type voltage-gated calcium channel (Ca_V_1.3). Mutant *CACNA1D* channels show activation at more hyperpolarized membrane potentials and, in some cases, reduced channel inactivation compared to wild-type channels (45). In line with the notion that these effects will lead to increased calcium entry, expression of mutant *CACNA1D* channels causes increased aldosterone production in the adrenocortical cancer cell line H295R (46). Again, similar to *KCNJ5* variants, additional evidence for a role of *CACNA1D* in PA came from the discovery of germ line variants at the same positions found to be mutated in tumors (45). Among 100 unrelated subjects with early-onset PA and hypertension, two carried *de novo* mutations in *CACNA1D*. Interestingly, these subjects had a multi-organ phenotype, including primary aldosteronism, seizures, and neurologic abnormalities (PASNA) (45). The discovery of mutations in calcium channels as a cause of PA may suggest that specific calcium channel blockers could be useful in patients carrying such mutations (46).

## ATPase Mutations in Primary Aldosteronism

Additional somatic mutations in APAs without corresponding germ line mutations have been identified. Beuschlein et al. first described heterozygous or hemizygous somatic mutations in the *ATP1A1* and *ATP2B3* genes in 5.2 and 1.6% of APAs, respectively. *ATP1A1* encodes a sodium/potassium ATPase subunit, whereas *ATP2B3* encodes the plasma membrane calcium ATPase. Mutations in both ATPases cluster within the M4 helix, again suggesting a gain-of-function mechanism (47). Azizan et al. subsequently demonstrated that *ATP1A1* mutations cause an ouabain-sensitive, voltage-dependent inward Na^+^ or H^+^ current, respectively. Heterologous expression of mutant *ATP1A1* in human adrenocortical H295R cells led to increased aldosterone production and *CYP11B2* expression levels (35), consistent with a role of mutant *ATP1A1* in cellular depolarization and activation of voltage-gated calcium channels, as with mutated *KCNJ5*. Similarly, a mutation in *ATP2B3* was shown to induce a pathological Na^+^ permeability, with increased intracellular Ca^2+^ levels and aldosterone production in H295R cells (48).

## Specific Features and Origin of *CACNA1D*- and Atpase-Mutant APAs

Azizan and colleagues first suggested an association of *CACNA1D* and *ATP1A1* mutations with a glomerulosa-like phenotype (35), whereas other groups reported mixed histological phenotypes (23, 36). Glomerulosa-like features in *CACNA1D* and *ATP1A1*-positive tumors could suggest that these tumors are derived from zona glomerulosa cells. Indeed, Nishimoto et al. recently studied 42 normal adrenal glands from kidney donors and identified so-called aldosterone-producing cell clusters (APCCs), nests of cells just below the adrenal capsule that feature high expression of aldosterone synthase and protrude into cortisol-producing cells (49). Remarkably, targeted next-generation sequencing of DNA from 23 APCCs identified known somatic *CACNA1D* mutations in six cases and known somatic *ATP1A1* mutations in two cases, suggesting that APCCs may represent precursors of a subtype of APAs. These results also support the presence of APCCs and potentially subclinical PA in a substantial number of apparently healthy individuals, which is interesting, given that prior clinical studies identified a higher risk of developing hypertension in individuals with increased aldosterone levels within the physiologic range (50). No somatic *KCNJ5* mutations were identified in APCCs, suggesting that APAs carrying such mutations may arise from cells of the zona fasciculata or may grow more rapidly, with precursors evading detection in apparently healthy individuals.

## Investigations of Multinodular Tumors

Even though the classical presentation of aldosterone-producing adenoma is that of a uninodular lesion, many cases feature associated hyperplasia or multiple secondary nodules, many of which do not show increased expression of aldosterone synthase. Investigations of individual nodules revealed the presence of characteristic APA mutations in aldosterone-producing nodules, whereas non-producing nodules do not carry such mutations (51). Some individuals carry different aldosterone-driver mutations in different nodules, suggesting that independent mutation events account for the development of multiple nodules (52, 53). Whether germ line susceptibility variants promote the formation of multiple tumors remain to be determined. Interestingly, some adenomas appear to show intra-tumoral heterogeneity, indicating that the somatic events underlying APA formation can also occur in the context of preexisting nodules (53). This has led to the proposal of a two-hit model of adenoma development, with one hit being responsible for proliferation and another hit causing hormone production (54). However, the rarity of such findings and the absence of second hits explaining proliferation in the exomes of tumors carrying aldosterone-driver mutations suggest that APA driver mutations alone are sufficient to cause proliferation and hormone production in the majority of APAs.

## *CACNA1H* Mutations in Familial Hyperaldosteronism

One additional ion channel gene implicated in PA to date has been found to be mutated in the germ line only, but not in APAs. A novel germ line heterozygous variant in the *CACNA1H* gene (M1549V) was found in 5 of 40 unrelated subjects with PA and hypertension diagnosed at age 10 or below (55). Microscopic glomerulosa hyperplasia without macroscopic enlargement was demonstrated in one subject who had undergone unilateral adrenalectomy, suggesting a limited proliferative effect of the variant. *CACNA1H* encodes the low-voltage-activated T-type calcium channel Ca_V_3.2 (56). Ca_V_3.2 has been hypothesized to be responsible for fine adjustments in the aldosterone production when activated by small changes in potassium or ATII levels and appears to be necessary for glomerulosa membrane potential oscillations (55, 57, 58). The observed M1549V variant causes impaired channel inactivation and a slight shift of activation to more hyperpolarized potentials (55), as well as increased *CYP11B2* expression (59), suggesting a pathophysiology similar to that of *CACNA1D* variants.

## Somatic Mutations in Adrenal Cushing’s Syndrome

Cushing’s syndrome features hypercortisolism and is associated with a plethora of signs and symptoms, including weight gain, hypertension, diabetes mellitus, lethargy, acne, depression, hirsutism, and increased mortality (60, 61). CPAs are less frequent than ACTH-secreting pituitary tumors (62), but still account for up to 10% of endogenous CS (60, 63, 64). Somatic *PRKAR1A* loss-of-function mutations were identified as a cause of sporadic CPAs in a hypothesis-driven approach (65).

Following the description of somatic mutations in PA, using exome sequencing, four groups independently identified somatic mutations in the *PRKACA* gene as a cause of CS (66–69). *PRKACA* encodes the catalytic subunit of protein kinase A involved in the regulation of adrenal cortisol production (see Introduction and Figure 1). Beuschlein and colleagues sequenced the exomes of 10 CPAs and identified heterozygous somatic *PRKACA* mutations in eight, with a frequency of 37% in the entire cohort of CPAs associated with overt CS. No *PRKACA* variants were found in CPAs associated with subclinical CS, APAs, or inactive adenomas, and the presence of *PRKACA* variants was associated with a more severe phenotype (66). All but one tumor carried a single variant, L206R, suggesting a gain-of-function effect. L206 is located in the highly conserved interaction site between the regulatory and the catalytic subunits of PKA, and binding of the regulatory subunit at this position prevents substrate phosphorylation. Molecular modeling and functional analysis of PKA activity suggested that the L206R mutation would lead to a steric hindrance and prevent inhibition of catalytic activity by the regulatory subunit (70). Somatic *PRKACA* variants other than L206R are exceedingly rare (71). Further support for the causative role of increased PKA activity in CS came from the discovery of germ line *PRKACA* duplications in subjects with bilateral adrenal hyperplasia and CS (66).

These results were confirmed in independent cohorts. Cao et al. reported an L205R variant (equivalent to L206R in the initial report) in the *PRKACA* gene in 27 of 39 CPAs. Further, two *GNAS* (G_αs_) mutations and a *CTNNB1* (β-catenin) mutation were found (see below) (67). Sato et al. screened tumors of 65 patients with ACTH-independent CS. They identified *PRKACA^L206R^* mutations in 52.3% and *GNAS* mutations in 16.9% of the tumors. In addition, they provided evidence of an association of *PRKACA^L206R^* with smaller tumor size and a more severe phenotype (68). Lastly, Goh et al. reported a *PRKACA^L206R^* mutation in 24% of CPAs (35% of cases with overt CS). They also reported *CTNNB1* mutations in 16% and *GNAS* mutations in 6% of tumors (69). Similar results were found in additional cohorts (39, 41, 71) (Table 1). Functionally, L206R has been shown to enhance the phosphorylation of PKA downstream effectors CREB and ATF in cell culture and tumor tissue samples (69) (Figure 1).

## Mutations in *GNAS* and *CTNNB1* in Cushing’s Syndrome and Primary Aldosteronism

Mutations in *GNAS* have long been known to inhibit GTPase activity of the G_αs_ subunit and thereby cause constitutive G_αs_ activation, abnormal cAMP signaling, endocrine hyperfunction, and tumor formation; postzygotic *GNAS* mutations are found in McCune–Albright syndrome, which can be associated with CS (72, 73). The discovery of mutually exclusive somatic gain-of-function mutations of *PRKACA* and *GNAS* in CPAs (see above) has further demonstrated that increased cAMP signaling is sufficient to cause tumorigenesis and cortisol hypersecretion. However, somewhat unexpectedly, given the absence of *PRKACA* mutations in APAs, *GNAS* variants were also reported in A/CPAs in two instances (42). On a molecular level, given the accessory role of ACTH in stimulating aldosterone secretion, increased cAMP signaling may play a role.

*CTNNB1* encodes β-catenin of the Wnt/β–catenin pathway, which is known to play an important role in adrenocortical development and cancer (74). Activating mutations are not only found in benign and malignant adrenal tumors (75) but also in tumors of other organs. Such mutations prevent β-catenin degradation and cause proliferation. Even though such events have been shown to trigger benign aldosterone-secreting and cortisol-secreting tumor development as well as malignancy in a mouse model and human tissue samples (36, 45, 69, 75–77), the exact mechanisms underlying hormone secretion in *CTNNB1* positive tumors remain to be determined.

In this context, a common pathway of *PRKACA, GNAS*, and *CTNNB1* has been suggested (78). However, it has been shown that *GNAS* and *CTNNB1* mutations are not always mutually exclusive in CPAs, and that mutations of *CTNNB1* are also present in non-secreting adrenal tumors (79). A recent study described an association with pregnancy in two of three cases with APAs and *CTNNB1* mutations and suggested that the manifestation may be mediated by *CTNNB1*-induced LHCGR expression and increased LH levels in pregnancy (80). However, the absence of an association with pregnancy in previously described female cases (81), the high prevalence of LHCGR overexpression in APAs (82), and the finding of *CTNNB1* mutations in male individuals with APAs (83) suggest a role of additional factors.

## Conclusion and Open Questions

Taken together, the recent findings on the genetic causes of APAs and CPAs suggest that both result from gain-of-function mutations that concurrently lead to excess hormone hypersecretion and increased proliferation. In most cases, a single mutation is apparently sufficient for tumor formation and hormone hypersecretion. There is little overlap between CPAs and APAs in terms of the mutational spectrum. While CPAs often carry mutations that lead to increased intracellular cAMP levels, mutations known to cause APAs mostly affect intracellular calcium signaling. Overlapping roles in the function of transcription factors ATF and CREB in glomerulosa and fasciculata function, as well as overlapping roles of signaling downstream of ACTH and calcium in cortisol and aldosterone synthesis, may explain the presence of *KCNJ5* and *GNAS* mutations in tumors secreting both cortisol and aldosterone (Figure 1). Open questions include the determinants of the histological phenotype of APAs with certain mutations, the molecular pathways involved in proliferation of both APAs and CPAs, potential additional factors that drive hormone production in tumors with *CTNNB1* mutations, and the pathogenesis of tumors without mutations in known driver genes. In summary, despite significant progress over the past few years, the pathophysiology behind CPAs and APAs has not been fully unraveled. Distinct and common molecular switches appear to exist in both disorders.

## Author Contributions

All authors listed have made substantial, direct, and intellectual contribution to the work and approved it for publication.

## Conflict of Interest Statement

The authors declare that the research was conducted in the absence of any commercial or financial relationships that could be construed as a potential conflict of interest.
